# The metabolome of fecal extracellular vesicles in patients with malignant solid tumors

**DOI:** 10.1038/s41598-025-14250-2

**Published:** 2025-08-11

**Authors:** Surbhi Mishra, Arina Maltseva, Anni I. Nieminen, Mikael Niku, Sonja Karikka, Jenni Hekkala, Sirpa Leppä, Pia Vihinen, Kaisa Sunela, Jussi Koivunen, Arja Jukkola, Ilja Kalashnikov, Päivi Auvinen, Okko-Sakari Kääriäinen, Juha Saarnio, Sanna Meriläinen, Tero Rautio, Raila Aro, Reetta Häivälä, Peeter Karihtala, Terhi Ruuska-Loewald, Justus Reunanen

**Affiliations:** 1 Research Unit of Clinical Medicine, University of Oulu, Oulu, Finland; 2 Biocenter Oulu, University of Oulu, Aapistie 5, P.O. Box 5281, Oulu, 90014 Finland; 3 Department of Veterinary Biosciences, Faculty of Veterinary Medicine, University of Helsinki, Helsinki, Finland; 4 Helsinki Metabolomics Center, Stem Cell and Metabolism Research Program, Faculty of Medicine, University of Helsinki, Helsinki, Finland; 5 Laboratory of Developmental Biology, Disease Networks Research Unit, Faculty of Biochemistry and Molecular Medicine, University of Oulu, Oulu, Finland; 6 Research Unit of Translational Medicine, University of Oulu, Oulu, Finland; 7 Department of Oncology, Helsinki University Hospital Comprehensive Cancer Center, University of Helsinki, Helsinki, Finland; 8 FICAN West Cancer Centre and Department of Oncology, Turku University Hospital and University of Turku, Turku, Finland; 9 Finnish Medicines Agency, Tampere, Finland; 10 Department of Medical Oncology and Radiotherapy and Medical Research Center, Oulu University Hospital and University of Oulu, Oulu, Finland; 11 Department of Oncology, Tampere Cancer Center, Faculty of Medicine and Health Technology, Tampere University Hospital, Tampere University, Tampere, Finland; 12 Research Program Unit, Applied Tumor Genomics, Faculty of Medicine, University of Helsinki, Helsinki, Finland; 13 Cancer Center, Kuopio University Hospital, Northern Savonia Healthcare Municipality, Kuopio, Finland; 14 Translational Medicine Research Unit, Medical Research Center Oulu, Oulu University Hospital, University of Oulu, Oulu, Finland; 15 Department of Pediatrics and Adolescent Medicine, Oulu University Hospital, Oulu, Finland

**Keywords:** Gut microbiome, Extracellular vesicles, Metabolome, Cancer, Small-molecule metabolites, Mass spectrometry, Biochemistry, Cancer, Microbiology, Molecular biology, Diseases, Translational research

## Abstract

Dysregulated metabolism, a hallmark of cancer, creates unique metabolic features that can be employed to elucidate cancer prognosis, personalized treatment, and therapeutic response. Metabolomics has emerged as a powerful tool for profiling biomarkers in cancer studies. Most cancer metabolomic research on extracellular vesicles (EVs) has focused on human biofluids as samples. The metabolome of fecal EVs, a connecting link for host-microbiome interactions in cancer, has not been extensively studied. In this controlled study, we investigated the metabolomic signatures of fecal EVs in patients with solid tumors. Fecal samples were collected from adult patients with solid tumors (*n* = 28) and healthy controls (*n* = 7). After the isolation of EVs from fecal samples, EV metabolites were identified using targeted metabolomics profiling based on liquid chromatography-mass spectrometry (LC-MS). The metabolomic profiles of the fecal EVs from both patients and controls were compared using R and Metabolite Set Enrichment Analysis was done using Metaboanalyst 6.0. The metabolomic profiles of fecal EVs showed several differences between patients with solid tumors and control subjects. L-glutamic acid was identified as the most significantly enriched metabolite in patients with solid tumors. Conversely, guanine and N-acetylneuraminate were the most significantly depleted metabolites in the fecal EVs of these patients. Metabolite Set Enrichment Analysis linked the identified EV metabolites to key metabolic pathways, including arginine biosynthesis, glyoxylate and dicarboxylate metabolism, and the biosynthesis of branched-chain amino acids and unsaturated fatty acids. Receiver operating characteristic (ROC) revealed that glutamic acid is the most effective metabolite in distinguishing cancer patients from healthy controls. Some of these metabolites may also have plausible bacterial origins, as described in previous studies. Distinct metabolic phenotypes were identified in patients with solid tumors by analyzing fecal EVs in this study. The metabolomic profiling of fecal EVs offers valuable insights into the interactions between the gut microbiome and the host as well as unique metabolic snapshot of the disease status in the context of cancer. Thus, fecal EVs should be included in advanced multi-omics analyses of cancer research, alongside other human biofluids.

**Supplementary Information:**

The online version contains supplementary material available at 10.1038/s41598-025-14250-2.

## Background

Extracellular vesicles (EVs) are nano-sized, spherical, and lipid bilayer-delimited particles secreted by live cellular organisms such as eukaryotes, bacteria and archaea. They contain a cargo of parent cell-derived bioactive substances such as DNA, RNA, proteins, and metabolites^1^ that can participate in diverse physiological processes in a biological system^2^. Consequently, EVs and their cargo may play a vital role in intercellular and interkingdom communication, including microbiome and host interactions associated with cancer pathogenesis^3,4^. Using omics technologies has allowed researchers to identify and study the bioactive cargo of extracellular vesicles of both host and bacterial origin^5–7^.

Metabolites, the final downstream products of protein translation and gene transcription or cellular perturbations to the proteome, genome or transcriptome^8,9^, link the genetic alterations of cancer cells to the overall disease phenotype^10,11^. Furthermore, the metabolites found in human biofluids provide insight into how cancer cells adapt to various pathophysiological stimuli, such as nutrient scarcity, hypoxia, or therapy, at specific time points^8^. Systemic biofluids like plasma, serum, urine, saliva, and sweat, which can be obtained non-invasively, have been extensively used in metabolomic studies. However, EVs have been less explored in this context. The key difference between metabolites in biofluids and those encapsulated within EVs is the protective environment that EVs provide. This shielding helps preserve the stability and functionality of metabolites, making EVs a more reliable source for insights into biological processes^12^. Moreover, EVs can traverse biological barriers and deliver their concentrated cargo to target cells throughout the body^13,14^.

The metabolomes of EVs may serve as biomarkers in cancer patients, as evidenced by studies on plasma-derived EVs from patients with endometrial cancer^15^, melanoma^16^, and breast cancer^17^, as well as urine-derived EVs from patients with prostate cancer^18^, colorectal cancer^19^, and lung cancer^20^. Feces are a significant source of EVs derived from the host and its diverse gut microbiota, highlighting the intricate connection between our bodies and the microorganisms that inhabit them^4,21^. However, only a limited number of studies have investigated the metabolome of EVs derived from feces^7^.

Our goal was to explore the metabolome of feces-derived EVs in patients with solid tumors in an observational, controlled clinical study. Specifically, we aimed to identify a potential panel of metabolites carried by fecal EVs that could be relevant to tumorigenesis and serve as biomarkers for cancer.

## Methods

### Study design and research subjects

This controlled study has been approved by the Helsinki University Hospital District Regional Committee on Medical Research Ethics (HUS/1377/2020) and Oulu University Hospital Ethical Committee, Finland (EETTMK 12/2020). The study was conducted in accordance with the Declaration of Helsinki. Two groups of participants, patients with advanced solid tumors (*n* = 28, including patients with non-small cell lung cancer, melanoma, renal cell carcinoma, urothelial carcinoma and head neck squamocellular carcinoma) and healthy controls (*n* = 7), were recruited, as described in our previous study^4^. All study participants gave their written informed consent. Fecal samples were self-collected by the study participants, transported to the research facility, and stored at − 80 °C. The clinical characteristics of study participants are listed in Supplementary Table 1.

### Isolation and characterization of fecal EVs

EVs were isolated from the feces of both solid tumors patients and healthy controls following the procedure described in our previous study^4^, according to the guidelines of Minimal information for studies of extracellular vesicles (MISEV2023)^22^. Briefly, each fecal sample was suspended in sterile phosphate-buffered saline (PBS), centrifuged at 14,000 × g for 30 min at 4 °C and filtered through a 40 μm nylon filter and 0.45 μm polyethersulfone (PES) filter to remove foreign particles and impurities. The purified samples were concentrated in Amicon^®^ Ultra-15 centrifugal filter units (Millipore, #UFC910024) by centrifugation, followed by the isolation of EVs using density gradient ultracentrifugation. Transmission electron microscopy (TEM) was used to characterize the morphology of isolated EVs. The concentration and size distribution of isolated EVs were measured using nanoparticle tracking analysis (NTA)^4^. Fecal EV preparations were stored at − 20 °C. We have submitted all relevant data of our experiments to the EV-TRACK knowledgebase (EV-TRACK ID: EV250039)^23^.

### Liquid chromatography-mass spectrometry analysis of fecal EV metabolites

Fecal EV metabolites were identified using a targeted, relative profiling method with an in-house standard library (RT and m/z). For the extraction of metabolites, 100 µl EV preparations were thawed on ice and transferred into 1.5 ml Eppendorf tubes along with 400 µl cold LC-MS grade extraction solution (Acetonitrile: Methanol: Milli-Q water; 40:40:20). Samples were subjected to vortexing for 2 min, sonication for 1 min and then centrifugation for 5 min at 14,000× *g* and 4 °C. The supernatants were collected in polypropylene tubes and dried in a nitrogen gas evaporator. The dried samples were suspended in 40 µL of extraction solvent (ACN: MeOH: MQ; 40:40:20) and vortexed for 2 min before being transferred into HPLC glass autosampler vials. 2 µL of samples were injected into a Thermo Vanquish UHPLC, coupled with a Q-Exactive Orbitrap quadrupole mass spectrometer (full scan, range: 55–825 MS1: m/z and RT from inhouse standard library) that was equipped with heated electrospray ionization (H-ESI) source probe (Thermo Fisher Scientific, Waltham, MA, USA). A SeQuant ZIC-pHILIC (2.1 × 100 mm, 5 μm particle) column (Merck, Darmstadt, Germany) was used for chromatographic separation. The gradient elution was performed using 20 mM ammonium hydrogen carbonate (pH 9.4) with a flow rate of 0.100 mL/min. Ammonium solution (25%) was used as mobile phase A and acetonitrile as mobile phase B. The gradient elution was initiated from 20% mobile phase A and 80% of mobile phase B and maintained till 2 min., followed by 20% mobile phase A gradually increasing up to 80% till 17 min., then 80–20% mobile phase A decrease in 17.1 min., and maintained up to 24 min. The instrument was controlled using Xcalibur 4.1.31.9 software (Thermo Fisher Scientific, Waltham, MA, USA). The quality of the LC-MS runs was monitored by injecting pooled quality control (QC) samples made of EV samples and blank samples (extraction buffer) between the samples. The data was prefiltered for background (> 20% peak intensity in blank/sample) and response standard deviation (% coefficient of variation within pooled QC).

### Metabolomic data analysis

The exported intensity data was analyzed using R (version 4.4.1)^24^. Missing values were imputed using k-nearest neighbor averaging^25^ if a metabolite was detected in more than 50% of samples within each study group. All other missing values were classified as zeros (missing in 50% or more samples within each study group). Metabolomic data were log-transformed, and quantile normalized (limma package)^26^. The significance of the solid tumors effect on the EV metabolome was tested using nonparametric permutational multivariate analysis of variance (perMANOVA)^27^ with 9,999 permutations (vegan package)^28^. The metabolomes were visualized as a general heatmap based on normalized metabolite abundances with the ComplexHeatmap package^29^, a plot after nonmetric multidimensional scaling (nMDS) with the vegan package^28^ and a dendrogram of unweighted pair group method with arithmetic mean (UPGMA) plotted with the dendextend package^30^ based on Euclidean distances among the samples. Branch support was assessed by approximately unbiased (AU) p-values using multiscale bootstrap resampling^31^ with 5,000 iterations in the pvclust package^32^; this number of iterations ensured accurate estimation of AU p-values (standard errors were less than 0.05).

The moderated t-test^33^ in the limma package^26^ was applied to assess the significance of abundance change of individual metabolites. The moderated t-test is more powerful than an ordinary t-test because it moderates the sample variances for each metabolite using the information on the distribution of sample variances across all metabolites with the help of an empirical Bayes method. The logFC and adjusted p-values were visualized as a volcano plot with the ggplot2 package^34^. Normalized abundances of significantly different metabolites were plotted as heatmaps with the ComplexHeatmap package^29^.

Metabolite Set Enrichment Analysis (MSEA) was conducted using MetaboAnalyst 6.0^35^ to identify biologically meaningful patterns among the identified fecal EV metabolites. The Over Representation Analysis (ORA) algorithm of MSEA was used, with a list of important metabolites as input data, which was then compared against 80 metabolite sets based on the Kyoto Encyclopedia of Genes and Genomes (KEGG)^36^ human metabolic pathways. The ORA was performed using the hypergeometric test to assess whether a particular metabolite set was overrepresented in the compound list beyond what would be expected by chance. One-tailed p-values were provided and adjusted for multiple comparisons.

We assessed the diagnostic potential of metabolites for distinguishing between healthy individuals and tumor patients using a receiver operating characteristic (ROC) analysis ^37^. The ROC curves, which plot sensitivity against (1-specificity), were generated for each metabolite significantly different between the analysed groups. The area under the curve (AUC) was calculated to quantify the overall diagnostic performance of the candidate metabolite. The analysis was done with the pROC package^38^.

## Results

The morphology of fecal EVs characterized using TEM and their size distribution analyzed with NTA were reported in our earlier study^4^. The median particle size of fecal EVs was 203.2 nm (IQR = 33.3) in patients with solid tumors and 157.9 nm (IQR = 37.9) in healthy controls.

We then characterized and compared the metabolome of fecal EVs isolated from solid tumor patients (*n* = 28) and healthy controls (*n* = 7) (Supplementary Table 1). A total of 41 metabolites were identified by targeted relative LC-MS (Supplementary Table 2).

### Metabolome of fecal EVs

**Fig. 1:**
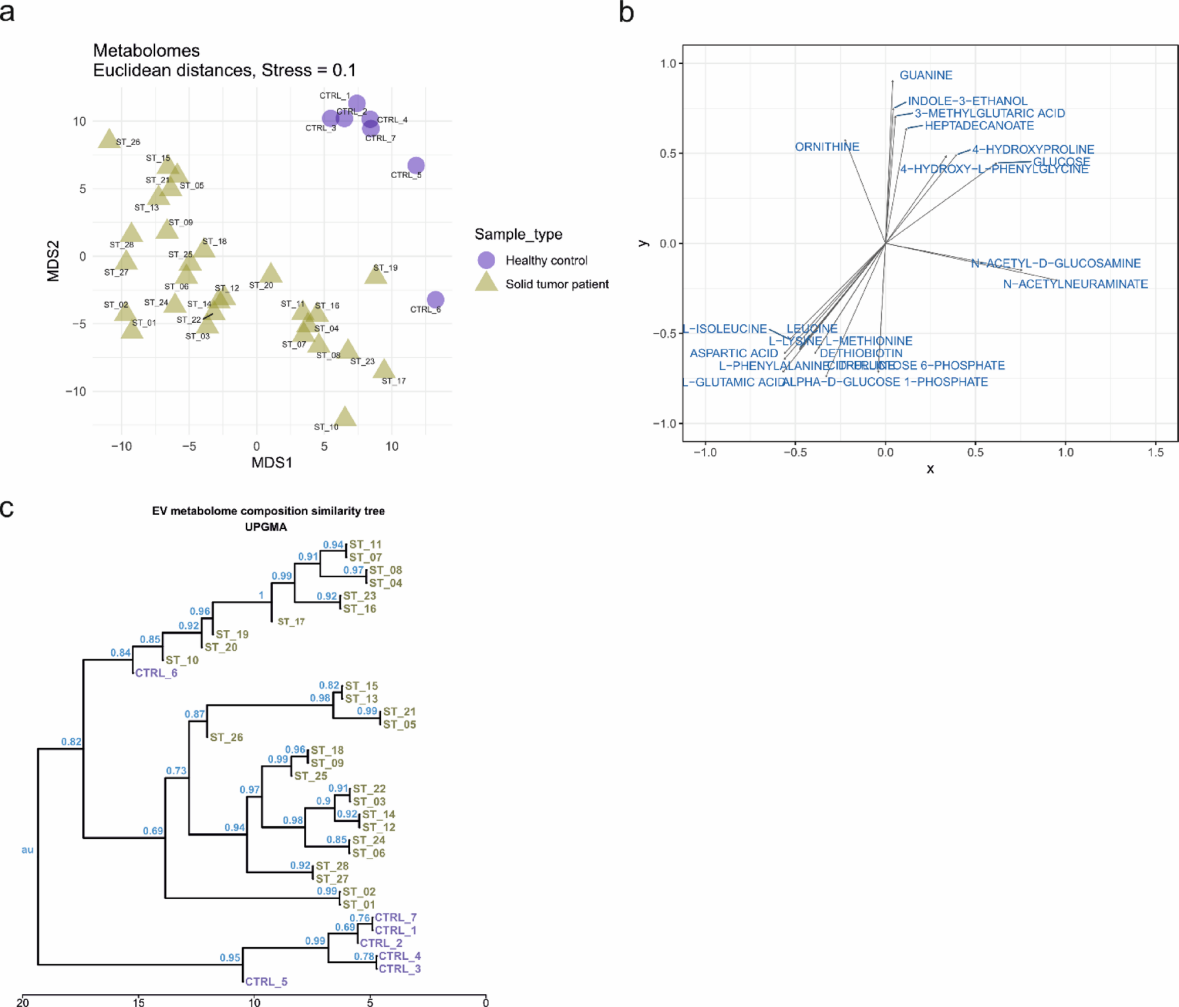
Visualization of fecal extracellular vesicle (EV) metabolome (**a**) Non-metric Multi-Dimensional Scaling (nMDS) ordination based on the Euclidean distance matrix, depicting the differences in the fecal EV metabolome between healthy controls and patients with solid tumors. (CTRL: Healthy controls; ST: Patients with solid tumors) (**b**) Plot depicting the metabolites with abundances significantly changing in the multidimensional space of analyzed samples and arrows showing the direction of abundance increase (**c**) Metabolome similarity tree constructed using Unweighted Pair Group Method with Arithmetic Mean (UPGMA) based on the Euclidean distance matrix (CTRL: Healthy controls; ST: Patients with solid tumors).

Fecal EVs from solid tumor patients and healthy controls differed in terms of their metabolome (Fig. 1). We visualized the fecal EV metabolomes using a heatmap to show metabolite abundances (Supplementary Fig. 1). We performed non-metric multidimensional scaling (nMDS) to ordain the samples based on a Euclidean distance matrix (Fig. 1a). Both visualizations demonstrated differences in the metabolome compositions between samples from solid tumor patients and healthy controls. A permutational multivariate analysis of variance (perMANOVA) showed the statistical significance of these differences (*p* < 0.001). Changes in the abundances of individual metabolites indicate that most metabolites differ between the patient and control groups (Fig. 1b). The metabolic diversity of fecal EVs between patients and controls was further supported by a metabolome similarity tree constructed using UPGMA (Fig. 1c).

**Fig. 2:**
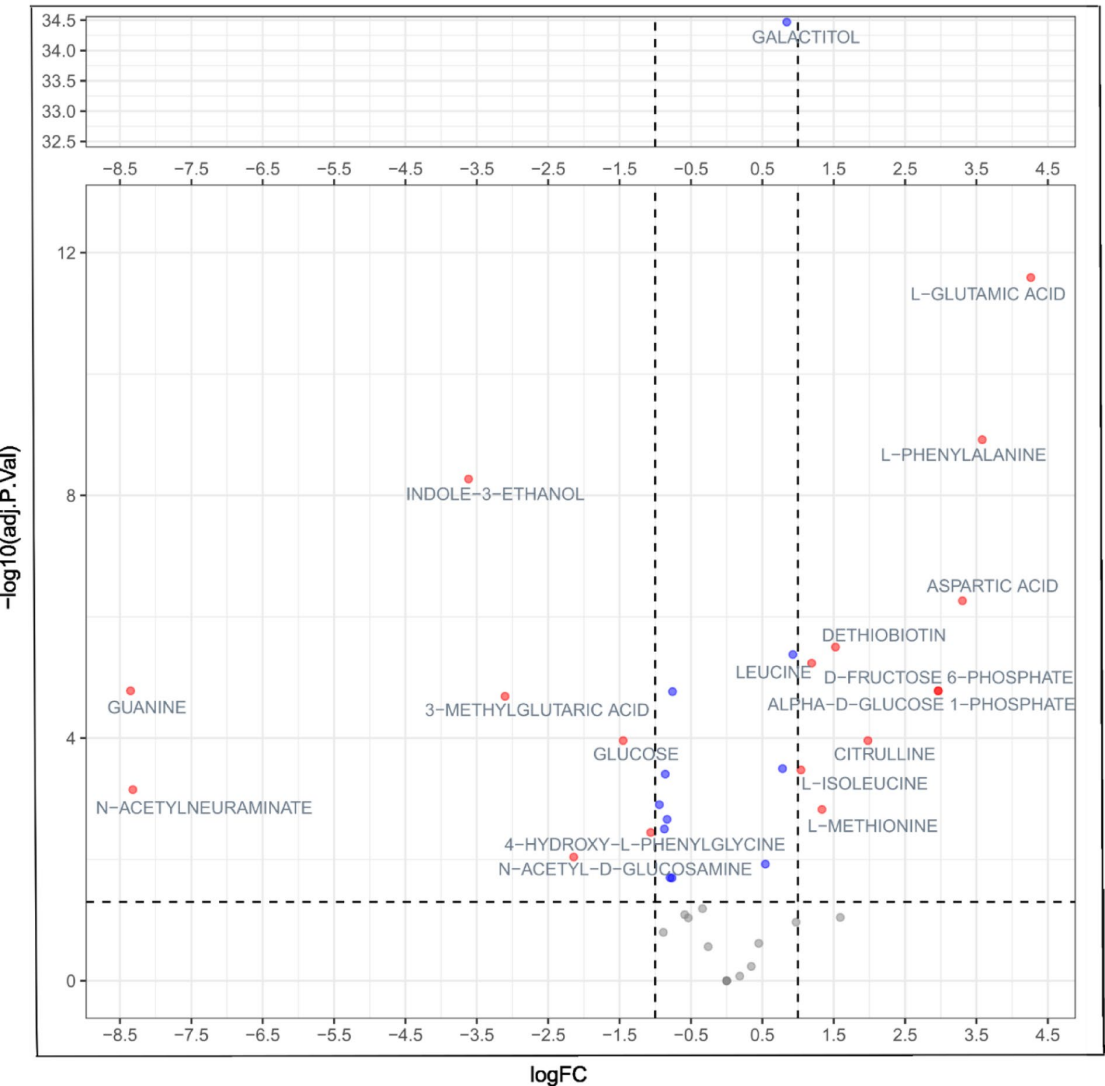
Volcano plot showing the abundance of fecal EV metabolites significantly different between healthy controls and patients with solid tumors. The X-axis indicates fold change, and the y-axis indicates adjusted p-value. The left part of the plot, i.e., negative fold change (FC) values, shows metabolites enriched in the healthy group; the right part, i.e., positive fold change (FC) values, shows metabolites enriched in the patients. Metabolites with FC equal to or more than two are orange dots, whereas those with less than two are blue dots. The horizontal dashed line on the plot shows a significance threshold (*p* = 0.05 after -log10 transformation), and the vertical dashed lines show a two-time increase (decrease) in metabolite abundance between groups in comparison.

A moderated t-test was performed to identify metabolites that exhibited a significant difference in abundance between the disease and control groups. Glutamic acid was the most significantly enriched metabolite in the fecal EVs of solid tumor patients compared to healthy controls. In contrast, guanine and N-acetylneuraminate were the most significantly depleted metabolites in these patients. Additional metabolites that showed increased abundance in solid tumor patients included phenylalanine, nicotinamide (NAM), aspartic acid, citrulline, alpha-D-glucose-1-phosphate, and D-fructose-6-phosphate. Conversely, the following metabolites were significantly depleted: indole-3-ethanol, 4-hydroxy-L-phenylglycine, N-acetyl-D-glucosamine, 3-methylglutaric acid, and glucose (Supplementary Table 2). The volcano plot illustrated the fold changes in metabolite distribution (Fig. 2).

### Metabolite set enrichment analysis

**Fig. 3:**
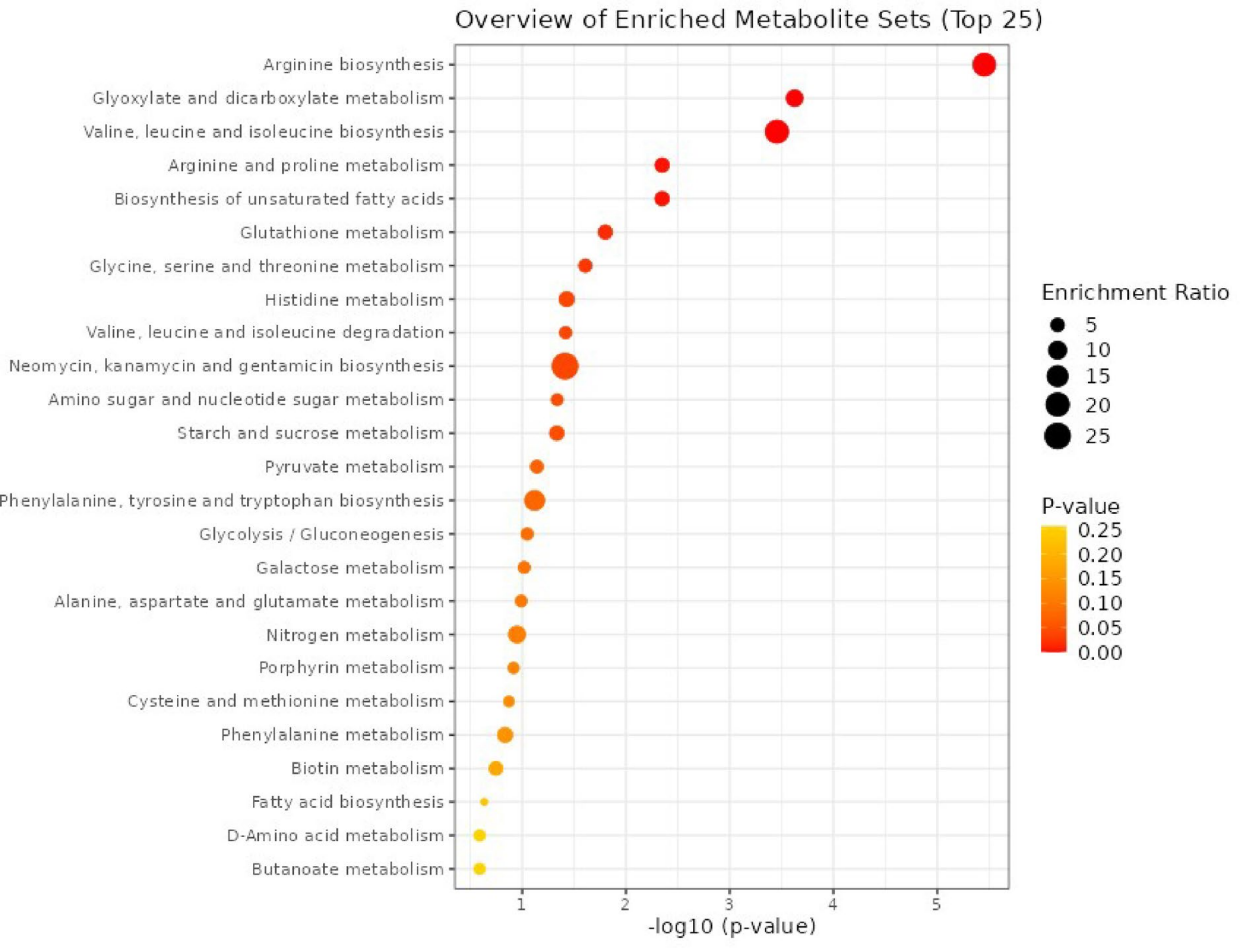
Metabolic Set Enrichment Analysis (MSEA) showing the overview of enriched metabolites sets in fecal extracellular vesicles (EVs): Metabolite sets enriched in fecal EVs were determined by over representation analysis (ORA) using the hypergeometric test (MetaboAnalyst 6.0). KEGG human metabolic pathways containing at least 5 entries were used as a metabolite set library. The metabolite sets are ranked according to the p-value, and color intensity from yellow to red indicates increasing statistical significance. The top 25 metabolite sets were demonstrated in the bar charts. The enrichment ratio represents the ratio of observed hits in the present data sets to expected hits in KEGG metabolic sets.

We performed metabolite set enrichment analysis (MSEA) using metabolites detected in our study and 80 metabolic sets based on KEGG human metabolic pathways as the metabolite set library to determine the enrichment of biologically relevant metabolic patterns in fecal EVs. We found an enrichment of the metabolites associated with arginine biosynthesis, glyoxylate and dicarboxylate metabolism, valine, leucine, and isoleucine biosynthesis, arginine and proline metabolism, and the biosynthesis of unsaturated fatty acids (Fig. 3). The results from ORA analysis are reported in Supplementary Table 3.

### Receiver operating characteristic (ROC) analysis

We performed ROC analysis to evaluate how effectively a selected metabolite distinguishes between cancer and control groups, calculating the Area Under Curve (AUC) values (Supplementary Table 4). ‘Sensitivity’ reflects the accuracy of disease detection, while ‘specificity’ indicates the precision of classifying control subjects. The top metabolites, shown in Table 1, demonstrated strong discriminatory abilities with high AUC values, confirming their effectiveness in differentiating between groups. Glutamic Acid (AUC = 0.98) emerged as the most predictive metabolite, followed by glucose (AUC = 0.97), nonanoate (AUC = 0.96) and guanine (AUC = 0.96). Sensitivity and specificity values close to 1 suggest high classification accuracy, while confidence intervals (AUC_lower and AUC_upper) further support the reliability of these findings.

**Table 1:** Receiver operating characteristic (ROC) analysis of fecal EV metabolites: area under curve (AUC)-based classification.

Metabolite	AUC	AUC_lower	AUC_upper	Sensitivity	Specificity
L-GLUTAMIC ACID	0.98469387755102	0.951139674416392	1	0.857142857142857	1
GLUCOSE	0.979591836734694	0.937166209458683	1	1	0.964285714285714
NONANOATE	0.964285714285714	0.9157144001572	1	1	0.928571428571429
GUANINE	0.961734693877551	0.902045230623096	1	0.857142857142857	0.964285714285714
DETHIOBIOTIN	0.948979591836735	0.879272485449785	1	1	0.857142857142857
ASPARTIC ACID	0.943877551020408	0.859953420591279	1	1	0.714285714285714
4-HYDROXYPROLINE	0.931122448979592	0.813442522404249	1	0.857142857142857	0.928571428571429
L-ISOLEUCINE	0.923469387755102	0.8195928062769	1	0.857142857142857	0.928571428571429
L-LYSINE	0.918367346938776	0.825927873082181	1	1	0.714285714285714
3-METHYLGLUTARIC ACID	0.915816326530612	0.749091623614268	1	0.857142857142857	1
L-PHENYLALANINE	0.915816326530612	0.748780921735335	1	0.857142857142857	1
4-HYDROXY-L-PHENYLGLYCINE	0.910714285714286	0.802524057990857	1	0.857142857142857	0.857142857142857
LEUCINE	0.903061224489796	0.711540642221971	1	0.857142857142857	1

## Discussion

In this observational, controlled clinical study, we investigated the metabolomic cargo of fecal EVs in patients with solid tumors using targeted metabolomic profiling. Our analysis revealed notable differences in the metabolome of fecal EVs between patients and controls. MSEA analysis showed that the metabolites identified from fecal EVs were associated with amino acid biosynthesis, unsaturated fatty acid biosynthesis, and glyoxylate and decarboxylate metabolism. Present results show the potential of fecal nanoparticles, i.e., EVs, secreted by host and gut microbiota, as significant components of the oncobiome.

Although cancer metabolomics research has primarily targeted EVs from blood serum or urine, there has been limited investigation into EVs derived from fecal samples. Fecal EVs contain components from both host and gut microbiota^4^. The gut microbiome plays a crucial role in shaping the host metabolism and influencing the production of metabolites that could be captured well in fecal EVs^7,39^. In two previous clinical studies in patients with colorectal cancer, metabolites of fecal EVs were suggested to be associated with gut microbiota and the pathogenesis of colorectal cancer^7,39^. Our study shows that the metabolome of fecal EVs may also play a significant role in patients with cancers other than colorectal cancer.

In our study, there was an enrichment of amino acids such as glutamic acid, phenylalanine, aspartate, and the aromatic alcohol indole-3-ethanol in fecal EVs from patients with solid tumors. These findings are consistent with an earlier study on patients with colorectal cancer ^7^. The metabolism of amino acids is often altered in cancer^40,41^. In our study, glutamate was the most significantly enriched and differentially abundant EV metabolite in patients with solid tumors, followed by phenylalanine and aspartate. Glutamate could influence cancer progression by regulating various cell signaling pathways in *ex vivo* and *in vitro* conditions^42,43^, and increased glutamate levels have been reported in cancerous tissues of patients with pancreatic ductal adenocarcinoma, breast cancer^44,45^ and prostate cancer^46^. In breast cancer research, phenylalanine has been found to convert into mutagenic, genotoxic, or carcinogenic compounds, such as phenols and indoles, in breast tissues^47^. Fecal metabolomic studies have also linked phenylalanine metabolism to gut dysbiosis in mice, showing its association with inflammation and oxidative stress^48^. Aspartate is central to cancer cell metabolism and is essential for protein and nucleotide biosynthesis and maintaining redox balance within the cancer cells^49^.

In the present study, we found that N-acetylneuraminate, N-acetyl-glucosamine, and indole-3-ethanol levels were significantly lower in the fecal EVs of solid tumor patients. N-acetylneuraminic acid, a predominant sialic acid, plays a key role in cellular interactions and has been identified as a tumor marker in the serum of patients with head and neck cancer^50^. Targeting sialic acids may effectively treat cancer, as shown by the tumor-inhibiting sialic acid mimetics *in vivo*^51^. Alterations in cell surface glycans play a critical role in cancer development. A study demonstrated that reducing glycosylation of O-linked β-N-acetyl glucosamine disrupts glutamine metabolism, resulting in a significant decline in cell proliferation and tumor growth in mouse models of pancreatic ductal adenocarcinoma^52^. Similarly, intraperitoneal injections of N-acetyl-D-glucosamine in a breast cancer xenograft model reduced cell proliferation and resulted in smaller tumors, less mitosis, and reduced angiogenesis compared to controls^53^. Indole-3-ethanol is a catabolite of tryptophan that may influence host physiology by being absorbed through the intestinal epithelium and entering the bloodstream, exhibiting anti-oxidative and anti-inflammatory properties^54^. Analysis of the human gut microbiome and cytokine responses in whole blood showed a negative correlation between interferon-gamma (IFNγ)^55^ production and bacterial genes that convert tryptophan into indole-3-ethanol, indicating that indole-3-ethanol may have anti-inflammatory properties^56^.

We performed a Metabolite Set Enrichment Analysis to categorize identified fecal EV metabolites using the KEGG database. The metabolites were associated with pathways including arginine biosynthesis, BCAA biosynthesis (valine, leucine, and isoleucine), and unsaturated fatty acid biosynthesis. Arginine, vital for tumor cell survival, is extensively consumed in tumor necrotic cores^57^. Dietary amino acids like proline and glutamine convert to citrulline in the intestines, which is then transformed into arginine in the bloodstream^58^. Tumors also utilize BCAAs for energy balance and nutrient signaling^59^. Alterations in glyoxylate and dicarboxylate metabolism have been observed in various cancer tissues^60^. Increased cellular metabolism during carcinogenesis enhances fatty acid production, which is linked to tumor viability and malignancy^61^.

In ROC analysis, glutamic acid was identified as a highly effective biomarker for differentiating between patients with solid tumors and healthy controls, followed by glucose, nonanoate, guanine, and dethiobiotin. These metabolites could assist in the early detection of diseases. Although the findings are encouraging, additional research with varied populations is needed to validate their reliability.

Research on the metabolic activities in the gut of healthy individuals has highlighted the relationship between the gut microbiome and fecal metabolites^62^. Certain metabolites found in our study have been linked to specific bacterial genera in previous studies. For instance, the bacterial genera *Ruminococcus*, *Dorea*, and *Blautia* have shown a positive correlation with the metabolite L-isoleucine. The metabolite L-leucine displayed a negative correlation with *Faecalibacterium*^63^. Additionally, the genus *Parabacteroides* was positively correlated with nicotinamide. Clostridium was positively associated with L-glutamate, while Acinetobacter was negatively correlated with it^64^. Furthermore, phenylalanine exhibited a positive correlation with unclassified genera from the families *Lachnospiraceae* and *Clostridiaceae*^65^. Various metabolites including L-aspartic acid, linoleic acid, L-isoleucine, L-lysine, L-phenylalanine, L-proline, L-tryptophan, L-tyrosine, and glutamic acid have been quantified from human feces and *E. coli* using Gas Chromatography/Mass Spectrometry^66^. The interplay between the metabolomes of the host and the gut bacteria, facilitated by bacterial EVs, could significantly influence health and disease status, including cancer pathogenesis^67^. Thus, profiling of gut-derived bacterial EVs becomes essential for cancer research, as they could provide a unique metabolic snapshot of the health/disease status.

The main strength of this study lies in its controlled design and the investigation of the metabolome of fecal EVs in patients with cancers other than colorectal cancer. Additionally, the metabolites found in fecal EVs may be particularly significant as they represent the final products of biological pathways. The EV matrix protects these metabolites, facilitating their systemic movement and concentrated delivery to target cells or tissues.

This study has some limitations. The sample size was relatively small, and we could not account for factors such as types of antibiotics used and dietary differences among patients. Antibiotic treatment can alter microbial diversity and balance, resulting in dysbiosis. This disruption may decrease beneficial bacteria like *Bifidobacterium* and *Eubacterium* while encouraging the growth of antibiotic-resistant strains^68^. The extent of these effects depends on factors such as the specific antibiotic used, its dosage, and the duration of treatment^69^. Due to limited information on the specific antibiotics used, we could not assess their impact on the gut microbiome and metabolomic profiles in cancer patients. The study included various cancer types, all of which were either locally advanced or metastatic, with localized cancers excluded. However, we could not evaluate how these tumor type variations affected the EV metabolome. As a pilot study, we could not establish a direct link between the metabolomic differences and cancer development or progression. The metabolomics profiling method did not detect all metabolites; therefore, another method could reveal more findings. However, further *in vitro* and *in vivo* studies using cell lines and animal models based on our findings will help clarify these associations. Additionally, the differential EV metabolites identified in our study could be further investigated for their potential as specific and accurate biomarkers for diagnosis, monitoring, risk prediction, and prognosis^8^.

## Conclusion

This observational, controlled clinical study showed that fecal EVs show differences in the metabolic phenotypes between patients with solid tumors and healthy controls. The significant metabolomic signatures identified using fecal EVs in patients with solid tumors provide valuable insights that could guide future targeted research. Our findings support the idea that metabolic signatures of fecal EVs should be included in oncobiome research as they could serve as non-invasive biomarkers for solid tumors, offering new possibilities for diagnostic and therapeutic approaches.

## Supplementary Information

Below is the link to the electronic supplementary material.

Supplementary Material 1

## Data Availability

The authors confirm that access restrictions apply to the data. The GDPR legislation requires us to protect the identity of participants, and the raw data cannot be publicly shared. The data generated and/or analyzed during the current study are available from the corresponding author on a reasonable request.

## Abbreviations

EVs: Extracellular vesicles
LC-MS: Liquid chromatography-mass spectrometry
DNA: Deoxyribonucleic acid
RNA: Ribonucleic acid
PES: Polyethersulfone
TEM: Transmission electron microscopy
NTA: Nanoparticle tracking analysis
H-ESI: Heated electrospray ionization
QC: Quality control
perMANOVA: Permutational multivariate analysis of variance
nMDS: Nonmetric multidimensional scaling
UPGMA: Unweighted pair group method with arithmetic mean
AU: Approximately unbiased
ORA: Over representation analysis
KEGG: Kyoto encyclopedia of genes and genomes
MSEA: Metabolite set enrichment analysis
ROC: Receiver operating characteristic
AUC: Area under curve
FC: Fold Change
NAM: Nicotinamide
Neu5Ac: N-acetylneuraminate
BCAA: Branched chain amino acids
O-GlcNAc: O-linked β-N-acetylglucosamine
IFNγ: Interferon-gamma
