# Supplementary material for: The metabolome of fecal extracellular vesicles in patients with malignant solid tumors
Source: Sci Rep. 2025 Aug 11;15:29402. doi: 10.1038/s41598-025-14250-2 (PMC12340066; doi:10.1038/s41598-025-14250-2)
Supplement: Supplementary file 1 — Supplementary Material 1 [file 41598_2025_14250_MOESM1_ESM.docx]

**SUPPLEMENTARY MATERIAL**

**Contents:**

1. Supplementary Figure 1
2. Supplementary Table 1
3. Supplementary Table 2
4. Supplementary Table 3
5. Supplementary Table 4


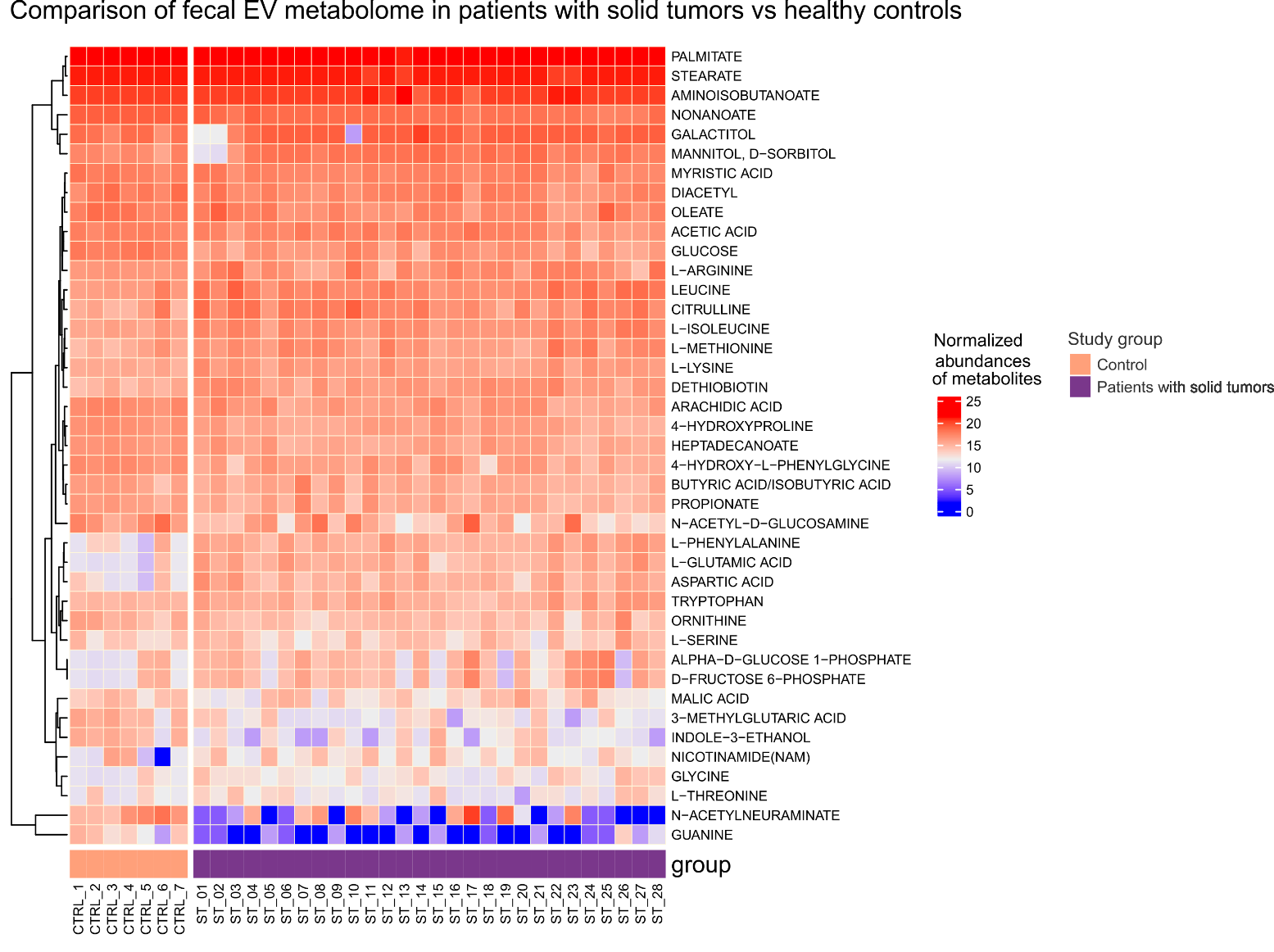


**Supplementary Figure 1.** Heatmap showing the normalized abundances of metabolites identified from fecal extracellular vesicles (EVs) of healthy controls and patients with solid tumors (CTRL: Healthy controls; ST: Patients with solid tumors)

**Supplementary Table 1: Clinical characteristics of study participants**

|  |  | Patients with Solid tumor | Healthy controls |
| --- | --- | --- | --- |
| Number of participants (*n)* |  | 28 | 7 |
| Sex | Male (%) | 64.29 | 42.86 |
|  | Female (%) | 35.71 | 57.14 |
| Age | Mean (SD) | 64.1 (9.5) | 57.4 (15.4) |
| Prior malignancy (%) |  | 10.7 | NA |
| Underlying diseases (%) | Cardiovascular diseases^*^  Pulmonary diseases^*^  Autoimmune diseases^*^ | 21.4  10.7  7.1 | NA  NA  NA |
| Recruits reporting antibiotic usage within the last 3 months (%) |  | 25 | No antibiotic usage |
| Tumor type/location n (%) | Non-small cell lung cancer | 15 (53.6) |  |
|  | Malignant melanoma | 5 (17.8) |  |
|  | Renal cell carcinoma | 5 (17.8) |  |
|  | Urothelial carcinomas | 1 (3.6) |  |
|  | Head and neck squamocellular carcinoma | 1 (3.6) |  |
|  | Other | 1 (3.6) |  |

**^*^**Requiring medication

**Supplementary Table 2: List of identified fecal EV metabolites**

| **S.No.** | **Fecal EV metabolite** | **Average expression**  **(fold change against the control group)** | **Adjusted P value** | **t*** |
| --- | --- | --- | --- | --- |
| 1 | 3-METHYLGLUTARIC ACID | -3.10458711039959 | 6.49461108189067e-06 | 12.3071944526726 |
| 2 | 4-HYDROXY-L-PHENYLGLYCINE | -1.06300875644132 | 0.00210292999021377 | 15.9008088267151 |
| 3 | 4-HYDROXYPROLINE | -0.942456624284678 | 0.00060959498733042 | 15.9374077474789 |
| 4 | ACETIC ACID | -0.258785601908652 | 0.240729541211737 | 17.2318668735167 |
| 5 | ALPHA-D-GLUCOSE 1-PHOSPHATE | 2.96461104754454 | 4.15643691674066e-06 | 13.7863241633508 |
| 6 | AMINOISOBUTANOATE | 5.46108297402621e-06 | 0.999433749320195 | 20.2939345670936 |
| 7 | ARACHIDIC ACID | -0.871686395617281 | 0.00176630557132613 | 16.3600710533596 |
| 8 | ASPARTIC ACID | 3.30429536040766 | 6.64861981702886e-08 | 14.4526077918388 |
| 9 | BUTYRIC ACID/ISOBUTYRIC ACID | -0.587234300655379 | 0.059524276138382 | 15.493286035483 |
| 10 | CITRULLINE | 1.97950689130735 | 4.01515887575802e-05 | 16.9856061329932 |
| 11 | DETHIOBIOTIN | 1.52557638460694 | 4.62053016626648e-07 | 16.0922357053354 |
| 12 | D-FRUCTOSE 6-PHOSPHATE | 2.96461104754454 | 4.15643691674066e-06 | 13.7863241633508 |
| 13 | DIACETYL | -0.537205537832582 | 0.07180330711793 | 17.3421550712016 |
| 14 | GALACTITOL | 0.842493836041161 | 8.28898703801928e-37 | 18.0627843008423 |
| 15 | GLUCOSE | -1.44954991822526 | 3.75207831500819e-05 | 16.5590660569106 |
| 16 | GLYCINE | 0.972727917786207 | 0.087018946335719 | 12.2951783026209 |
| 17 | GUANINE | -8.35114567771643 | 4.45975778586958e-06 | 6.16778411414175 |
| 18 | HEPTADECANOATE | -0.859065096197458 | 0.000172206199901035 | 15.8257948170622 |
| 19 | INDOLE-3-ETHANOL | -3.6158042462702 | 5.22968271041109e-10 | 11.7893918730906 |
| 20 | L-ARGININE | 0.450009317128337 | 0.205730353947625 | 16.7174296784861 |
| 21 | LEUCINE | 1.19129121337131 | 1.13650529757261e-06 | 17.2774927978553 |
| 22 | L-GLUTAMIC ACID | 4.26197468695896 | 1.24978124015471e-13 | 14.4342971686596 |
| 23 | L-ISOLEUCINE | 1.04197074588014 | 0.000138981090055887 | 16.6872720463523 |
| 24 | L-LYSINE | 0.78377400696994 | 0.000124279223419026 | 16.0024413610384 |
| 25 | L-METHIONINE | 1.33524259696549 | 0.000768001202898386 | 16.3394317967425 |
| 26 | L-PHENYLALANINE | 3.58165150725436 | 8.83078194644995e-11 | 14.7799811991831 |
| 27 | L-SERINE | 0.345894887669685 | 0.522662546277902 | 13.7258060310972 |
| 28 | L-THREONINE | 0.184514436139521 | 0.777524270504458 | 12.0096013894555 |
| 29 | MALIC ACID | -0.886769733732435 | 0.131904464746273 | 13.2546119946045 |
| 30 | MANNITOL, D-SORBITOL | 0.927769084952537 | 7.14730091312657e-07 | 17.3932276289913 |
| 31 | MYRISTIC ACID | -0.337853847461255 | 0.045857527317509 | 17.4442463806715 |
| 32 | N-ACETYL-D-GLUCOSAMINE | -2.14262046368407 | 0.0055546802075485 | 15.1089520503729 |
| 33 | N-ACETYLNEURAMINATE | -8.31905776150639 | 0.000327433638725016 | 9.96354017649946 |
| 34 | NICOTINAMIDE(NAM) | 1.59217167358155 | 0.0683890819522637 | 12.3920087859153 |
| 35 | NONANOATE | -0.757850817510144 | 5.01097462399993e-06 | 18.4711920216053 |
| 36 | OLEATE | -0.765437555732096 | 0.0137633071924982 | 17.3324685311359 |
| 37 | ORNITHINE | -0.793134117892225 | 0.0131503612651347 | 14.5228293209226 |
| 38 | PALMITATE | -2.84344780368104e-07 | 0.99996983482208 | 21.35826420526 |
| 39 | PROPIONATE | -0.833688535171762 | 0.00117086000892331 | 15.4383639921404 |
| 40 | STEARATE | -6.87838643267807e-06 | 0.999279507906708 | 21.0122350213647 |
| 41 | TRYPTOPHAN | 0.543211174677183 | 0.00756157792771664 | 15.2470930187042 |

^*^ t-value represents the M-value ratio (a measure of the difference between two groups) to its standard error

**Supplementary Table 3:** **Results from Over-Representation Analysis**

| **Metabolite set** | **Total** | **Expected** | **Hits** | **Raw p** | **Holm p** | **FDR** |
| --- | --- | --- | --- | --- | --- | --- |
| Arginine biosynthesis | 14 | 0.27 | 5 | 3.53E-06 | 2.82E-04 | 2.82E-04 |
| Glyoxylate and dicarboxylate metabolism | 31 | 0.60 | 5 | 2.37E-04 | 1.87E-02 | 9.36E-03 |
| Valine, leucine and isoleucine biosynthesis | 8 | 0.16 | 3 | 3.51E-04 | 2.74E-02 | 9.36E-03 |
| Arginine and proline metabolism | 36 | 0.70 | 4 | 4.48E-03 | 3.45E-01 | 7.17E-02 |
| Biosynthesis of unsaturated fatty acids | 36 | 0.70 | 4 | 4.48E-03 | 3.45E-01 | 7.17E-02 |
| Glutathione metabolism | 28 | 0.55 | 3 | 1.58E-02 | 1.00E+00 | 2.10E-01 |
| Glycine, serine and threonine metabolism | 33 | 0.64 | 3 | 2.46E-02 | 1.00E+00 | 2.81E-01 |
| Histidine metabolism | 16 | 0.31 | 2 | 3.72E-02 | 1.00E+00 | 3.09E-01 |
| Valine, leucine and isoleucine degradation | 39 | 0.76 | 3 | 3.81E-02 | 1.00E+00 | 3.09E-01 |
| Neomycin, kanamycin and gentamicin biosynthesis | 2 | 0.04 | 1 | 3.86E-02 | 1.00E+00 | 3.09E-01 |
| Amino sugar and nucleotide sugar metabolism | 42 | 0.82 | 3 | 4.60E-02 | 1.00E+00 | 3.09E-01 |
| Starch and sucrose metabolism | 18 | 0.35 | 2 | 4.63E-02 | 1.00E+00 | 3.09E-01 |
| Pyruvate metabolism | 23 | 0.45 | 2 | 7.21E-02 | 1.00E+00 | 4.33E-01 |
| Phenylalanine, tyrosine and tryptophan biosynthesis | 4 | 0.08 | 1 | 7.58E-02 | 1.00E+00 | 4.33E-01 |
| Glycolysis / Gluconeogenesis | 26 | 0.51 | 2 | 8.94E-02 | 1.00E+00 | 4.77E-01 |
| Galactose metabolism | 27 | 0.53 | 2 | 9.54E-02 | 1.00E+00 | 4.77E-01 |
| Alanine, aspartate and glutamate metabolism | 28 | 0.55 | 2 | 1.02E-01 | 1.00E+00 | 4.78E-01 |
| Nitrogen metabolism | 6 | 0.12 | 1 | 1.12E-01 | 1.00E+00 | 4.96E-01 |
| Porphyrin metabolism | 31 | 0.60 | 2 | 1.21E-01 | 1.00E+00 | 5.08E-01 |
| Cysteine and methionine metabolism | 33 | 0.64 | 2 | 1.34E-01 | 1.00E+00 | 5.35E-01 |
| Phenylalanine metabolism | 8 | 0.16 | 1 | 1.46E-01 | 1.00E+00 | 5.56E-01 |
| Biotin metabolism | 10 | 0.20 | 1 | 1.79E-01 | 1.00E+00 | 6.52E-01 |
| Fatty acid biosynthesis | 47 | 0.92 | 2 | 2.32E-01 | 1.00E+00 | 7.90E-01 |
| D-Amino acid metabolism | 15 | 0.29 | 1 | 2.57E-01 | 1.00E+00 | 7.90E-01 |
| Butanoate metabolism | 15 | 0.29 | 1 | 2.57E-01 | 1.00E+00 | 7.90E-01 |
| Nicotinate and nicotinamide metabolism | 15 | 0.29 | 1 | 2.57E-01 | 1.00E+00 | 7.90E-01 |
| Citrate cycle (TCA cycle) | 20 | 0.39 | 1 | 3.27E-01 | 1.00E+00 | 8.78E-01 |
| Fructose and mannose metabolism | 20 | 0.39 | 1 | 3.27E-01 | 1.00E+00 | 8.78E-01 |
| Pantothenate and CoA biosynthesis | 20 | 0.39 | 1 | 3.27E-01 | 1.00E+00 | 8.78E-01 |
| beta-Alanine metabolism | 21 | 0.41 | 1 | 3.40E-01 | 1.00E+00 | 8.78E-01 |
| Propanoate metabolism | 21 | 0.41 | 1 | 3.40E-01 | 1.00E+00 | 8.78E-01 |
| Pentose phosphate pathway | 23 | 0.45 | 1 | 3.66E-01 | 1.00E+00 | 9.16E-01 |
| Lipoic acid metabolism | 28 | 0.55 | 1 | 4.27E-01 | 1.00E+00 | 1.00E+00 |
| Lysine degradation | 30 | 0.58 | 1 | 4.49E-01 | 1.00E+00 | 1.00E+00 |
| Sphingolipid metabolism | 32 | 0.62 | 1 | 4.71E-01 | 1.00E+00 | 1.00E+00 |
| Fatty acid elongation | 38 | 0.74 | 1 | 5.31E-01 | 1.00E+00 | 1.00E+00 |
| Fatty acid degradation | 39 | 0.76 | 1 | 5.40E-01 | 1.00E+00 | 1.00E+00 |
| Tryptophan metabolism | 41 | 0.80 | 1 | 5.59E-01 | 1.00E+00 | 1.00E+00 |
| Primary bile acid biosynthesis | 46 | 0.90 | 1 | 6.01E-01 | 1.00E+00 | 1.00E+00 |
| Purine metabolism | 70 | 1.36 | 1 | 7.56E-01 | 1.00E+00 | 1.00E+00 |

**Supplementary Table 4: Receiver operating characteristic (ROC) analysis of fecal EV metabolites: Area under curve (AUC)-based classification**

| Metabolite | AUC | AUC_lower | AUC_upper | Sensitivity | Specificity |
| --- | --- | --- | --- | --- | --- |
| L-GLUTAMIC ACID | 0.98469387755102 | 0.951139674416392 | 1 | 1 | 0.857142857142857 |
| L-GLUTAMIC ACID | 0.98469387755102 | 0.951139674416392 | 1 | 0.857142857142857 | 1 |
| GLUCOSE | 0.979591836734694 | 0.937166209458683 | 1 | 1 | 0.964285714285714 |
| NONANOATE | 0.964285714285714 | 0.9157144001572 | 1 | 1 | 0.928571428571429 |
| GUANINE | 0.961734693877551 | 0.902045230623096 | 1 | 0.857142857142857 | 0.964285714285714 |
| DETHIOBIOTIN | 0.948979591836735 | 0.879272485449785 | 1 | 1 | 0.857142857142857 |
| ASPARTIC ACID | 0.943877551020408 | 0.859953420591279 | 1 | 1 | 0.714285714285714 |
| 4-HYDROXYPROLINE | 0.931122448979592 | 0.813442522404249 | 1 | 0.857142857142857 | 0.928571428571429 |
| L-ISOLEUCINE | 0.923469387755102 | 0.8195928062769 | 1 | 0.857142857142857 | 0.928571428571429 |
| L-LYSINE | 0.918367346938776 | 0.825927873082181 | 1 | 1 | 0.714285714285714 |
| 3-METHYLGLUTARIC ACID | 0.915816326530612 | 0.749091623614268 | 1 | 0.857142857142857 | 1 |
| L-PHENYLALANINE | 0.915816326530612 | 0.748780921735335 | 1 | 0.857142857142857 | 1 |
| 4-HYDROXY-L-PHENYLGLYCINE | 0.910714285714286 | 0.802524057990857 | 1 | 0.857142857142857 | 0.857142857142857 |
| LEUCINE | 0.903061224489796 | 0.711540642221971 | 1 | 0.857142857142857 | 1 |
| HEPTADECANOATE | 0.887755102040816 | 0.775916496673813 | 0.999593707407819 | 1 | 0.678571428571429 |
| L-METHIONINE | 0.88265306122449 | 0.720369145978838 | 1 | 0.857142857142857 | 0.857142857142857 |
| CITRULLINE | 0.875 | 0.671731886540412 | 1 | 0.857142857142857 | 0.928571428571429 |
| INDOLE-3-ETHANOL | 0.875 | 0.680981802384019 | 1 | 0.857142857142857 | 0.928571428571429 |
| MANNITOL, D-SORBITOL | 0.872448979591837 | 0.754851694806347 | 0.990046264377326 | 1 | 0.75 |
| ARACHIDIC ACID | 0.857142857142857 | 0.728006344198802 | 0.986279370086913 | 0.857142857142857 | 0.785714285714286 |
| GALACTITOL | 0.826530612244898 | 0.692811412671006 | 0.96024981181879 | 1 | 0.75 |
| N-ACETYLNEURAMINATE | 0.811224489795918 | 0.670870380406824 | 0.951578599185013 | 1 | 0.678571428571429 |
| PROPIONATE | 0.808673469387755 | 0.638390890800023 | 0.978956047975487 | 0.857142857142857 | 0.821428571428571 |
| N-ACETYL-D-GLUCOSAMINE | 0.798469387755102 | 0.652847610055149 | 0.944091165455055 | 1 | 0.642857142857143 |
| OLEATE | 0.775510204081633 | 0.598169373756926 | 0.95285103440634 | 1 | 0.464285714285714 |
| OLEATE | 0.775510204081633 | 0.598169373756926 | 0.95285103440634 | 0.571428571428571 | 0.892857142857143 |
| TRYPTOPHAN | 0.775510204081633 | 0.627185838622006 | 0.92383456954126 | 1 | 0.607142857142857 |
| ORNITHINE | 0.727040816326531 | 0.477525491917025 | 0.976556140736037 | 0.571428571428571 | 0.892857142857143 |
| ALPHA-D-GLUCOSE 1-PHOSPHATE | 0.704081632653061 | 0.481989221518528 | 0.926174043787595 | 0.714285714285714 | 0.821428571428571 |
| D-FRUCTOSE 6-PHOSPHATE | 0.704081632653061 | 0.481989221518528 | 0.926174043787595 | 0.714285714285714 | 0.821428571428571 |
